# List of reviewers

**DOI:** 10.2471/BLT.26.970126

**Published:** 2026-01-01

The Editorial Team would like to thank all those who gave generously of their time and expertise in reviewing the papers for the *Bulletin of the World Health Organization *in 2025. We apologize to anyone whose name has been inadvertently omitted from the following list:

## A

Abbasi, Ebrahim

Abbott, Frederick

Abdool, Karim Safura

Abeykoon, Palitha

Abimbola, Seye

Aboagye, Sammy

Addisu, Yohannes

Adewoyin, Yemi

Adeyemi, Ebenezer

Adjei, Michael

Adnan, Hassan Sami

Adoga, Moses

Afzali Harsini, Pooyan

Agache, Ioana

Akter, Shamima

Al Awaidy, Salah

Al Shorbaji, Najeeb

Alazab, Raed

Allen, Koya

Allipour-Birgani, Ramesh

Almeida, Joana

Alonso, Francisco

Aloosh, Mehdi

Alvargonzález, David

Alves da Costa, Filipa

Amico, Rivet

Amin, Nailah

Anderson, Michelle

Arab-Zozani, Morteza

Aran, Veronica

Arcury, Thomas

Aremu, Stephen

Asbu, Eyob

Askew, Ian

Askie, Lisa

Atroshchanka, Olga

Avalos, Miguel

Aye, Khin

Azadnajafabad, Sina

## B

Baek, Yeji

Bahena, Alejandro

Bailey, Helen

Balasubramanian, Chitra

Bartlett , Emily

Baskaran, Pritish

Baumann, Andrea

Begum, Shahnur

Bell, Samira

Bellingham-Young, Denise

Berezvai, Zombor

Bershteyn, Anna

Betran, Ana Pilar

Bhandari, Dinesh

Bhavsar, Vishal

Bhullar , Shilpy

Birchenall-Jiménez, Claudia

Bitanihirwe, Byron

Bitter, Cindy

Boerma, Ties

Bollars, Caroline

Bonfim, Camila

Boon, Heather

Borst, Robert

Boyce, Ross

Brito, Miguel

Brown, Paul

Brugger, Katharina

Bruno, Alfredo

## C

Cabral Schveitzer, Mariana

Campbell, F. Hanna

Cano, Jorge

Capewel, Simon

Cash-Gibson, Lucinda

Causevic, Sara

Cegolon, Luca

Cesari, Matteo

Chadha, Vineet

Chambers, Georgina

Chatterjee, Uma

Chaurasia, Akhilanand

Chen, Qi

Chiossi, Scott

Chipfakacha, Vitalis

Chute, Chrisptopher

Citron, Isabelle

Codyre, Patricia

Congdon, Nathan

Crainey, James

Cramer , Holger

Cumming, Toby

## D

Dalas, Shona

Das, Saibal

Das, Aparup

Dasgupta, Purnamita

Datema, Tjeerd

Datta, Manjula

Daube, Mike

Dawson, Walter

de Balogh, Katinka

De Sa, Thiago

de Witte, Luc

Dean, Elethia

DeJong, Jocelyn

Dekker, Marieke

Demarzo, Marcelo

Dempsey, Kathy

Deyno, Serawit

Diaz, Susan

Diaz, Theresa

Dol, Justine

Dolmazon, Virginie

Domenech de Cellès, Matthieu

Dong, Jicui

Dorj, Gereltuya

Downe, Soo

Dyer, Silke

## E

Ebi, Kristie

Ekzayez, Abdulkarim

El Idrissi, Ahmed

Eldaly, Sameh

Eliakimu, Eliudi

Elopre, Latesha

El-Saharty, Sameh

Elsharkawi, Hossam

Eze, Paul

## F

Fai, Karl Gwei Njuwa

Falzon, Dennis

Fasina, Folorunso

Faus, Mireia

Fene, Fato

Ferradini, Laurent

Figueras, Albert

Filippi, Veronique

Fletcher, Martin

Fonseca, Henrique

Fonseca, Valter

Ford, Nathan

Fraser, Joy

Frick, K Davina

Fryatt, Robert

## G

Gable, Lance

Galasinski, Darius

Gall, Alana

Gandra, Sumanth

Geissler, Alexander

Ghosh, Arnab

Ghosh, Kanjaksha

Ghosh, Rakesh

Ghoshal, Arun

Giri, Basant

Gleason, Kelsey

Godatwar, Pawan

Godura, Shefali

Goldblatt, Elizabeth

Gopinathan, Unni

Gray, Andrew

Gredebäck, Gustaf

Greenhalgh, Trish

Grépin, Karen

Griffiths, Elwyn

Gunawardane, Damitha

Gupta, Madhur

## H

HabibiSaravi, Reza

Haby, Michelle

Hall, Jean

Hannewinkel, Reiner

Hanson, Claudia

Hanssen, Odd

Haq, Zaeem

Haranath, Sai Praveen

Hartono, Risky

Harutyunyan, Vachagan

Hauk, Cathrin

He, Anqi

Henriques-Neto, Duarte

Holm, Michelle

Holzinger, Andreas

Homer, Caroline

Horsburgh, Bob

Hou, Chengbin

Housen, Tambri

Howell, Embry

Hull, Terence

Hunter, Jennifer

Husain, Noor

Huseynov, Shahin

Hussain, Tahziba

Huttner, Benedikt

## I

Ibanez, Agustin

Ijaz, Nadine

IJsselmuiden, Carel

Ikuomola, Felix

Ingelbeen, Brecht

Inyang, Ibanga

Irish, Seth

Irving, Adam

Islam, Zobida

Izidoro, Jans

## J

Jacobsen, Kathryn

Järvinen, Teppo

Jayaraj, Vivek

Jenei, Kristina

Jiao, Fei

Johler, Sophia

Johnson, Leigh

## K

Kahwaji, Areej

Källander, Karin

Kalyatanda, Gautam

Kam, Kai Man Joseph

Kamenshchikova, Alena

Kamradt-Scott, Adam

Kanda, Mikiko

Kaplan, Karyn

Karlinsky, Ariel

Kazeem, Olalekan

KC, Ashish

Kebede, Adane

Keenan, William

Keramat, Syed

Khorrami, Zahra

Kim, Sungchol

Koch, Marta

Kohno, Ayako

Kollipara, Aparna

Konduru, Laalithya

Kosmützky, Anna

Kovats, Sari

Kuruvilla, Shyama

Kutluk, Tezer

Kwaja, Chris

## L

Lahariya, Chandrakant

Laher, Beverly

Lal, Stuti

Larson, Heidi

Lau, Eric

Lawrence, Mark

Leontsini, Elli

Leung, Kathy

Lévy, Jacques

Lewis, Rosamund

Li, Qingfeng

Lima Cavaletti Guerra, Ana

Lin, Vivian

Littler, Katherine

Liu, Haopeng

Liu, Jianping

Liu, Qin

Liu, Xiaoyun

Loffreda, Giulia

Lokmic-Tomkins, Zerina

Long, Qian

Lopert, Ruth

Lund, Andrea

Luschin-Ebengreuth, Marion

## M

Madans, Jennifer

Maes, Kenneth

Mahat, Agya

Malme, Kristian

Marasini, Sabina

Mariotti, Silvio

Mascayano, Franco

Mawanda, James

McCoy, Matthew

McKay, Heather

McMonagle, Christine

McPherson, Cheryl

Meade, Robert

Mehboob, Gulrukh

Mendelson, Marc

Mierau, Jochen

Min, Kaiyuan

Minas, Iraklis

Mittal, Mahima

Mock, Charles

Mock, Nancy

Molyneux, Elizabeth

Montgomery, Maggie

Mora, Sara

Morais Nunes, Alexandre

Morton, Stephen

Mukherjee, Pulok

Murphy, Kelly

Murray, Colleen

Murray, Megan

Muschialli, Luke

## N

Nanyangwe-Moyo, Tina

Ndembi, Nicaise

Ndumba, Idah

Nessa, Ashrafun

Niazi, Sarfaraz

Nicholson, Emily

Nieto, Natalia

Ninot, Gregory

Nola, Iskra

Norris, Susan

## O

Obermann, Konrad

Occhipinti, Jo-An

O'Dougherty, Sheila

Okumagba, Mamodesan

Oliveira, Claudia

Olusanya, Bolajoko

Onarheim, Kristine

Orchard, Suzanne

Otukpa, Emmanuel

Ouoba, Kampadilemba

Ozanne-Smith, Joan

## P

Pachman, Joseph

Paez, Carolina

Paina, Ligia

Palacio-Mejia, Lina

Park, Hong-Jae

Parker, Courtney

Patel, Dipti

Pathak, Vaishnavi

Pega, Frank

Perez Gonzalez, Maria Mercedes

Perry, Henry

Perucic, Anne'Marie

Peter, Trevor

Pineau, Pascal

Polak, Paulina

Pope, Anne

Power, Jennifer

Pradhan, Rachita

Pratiti, Rebecca

Pust, Ronald

## Q

Queiroz, Bernardo

## R

Rai, Rajesh Kumar

Raja'a, Yahia

Rao, Neethi

Ratevosian, Jirair

Rathi, Megha

Ratnayake, Ruwan

Raventós Vorst, Henriette

Razum, Oliver

Reinoehl, Jennifer

Riboulet-Zemouli, Kenzi

Richards, Heather

Richards, Nicola

Riddell , Travis

Rietveld, Hans

Rittirong, Jongjit

Roberts, Charlee

Roberts, Teri

Rollins, Nigel

Ross, Jeremy

## S

Sabet, Farnaz

Sadana, Ritu

Sahu, Mahesh

Saidi, Sam

Saigí-Rubió, Francesc

Salazar, Miguel Antonio

Samad, Lubna

Sana, Hamaiyal

Sandall, Jane

Sant'Anna, Clemax

Santoso, Budiono

Sarkar, Swarup

Saunderson, Paul

Savage, Amy

Saxena, Abha

Sconza, Rebecca

Seeher, Katrin

Seifman, Richard

Selleck, Paul

Sen, Kasturi

Sevcikova, Petra

Shafik, Amal

Sharma, Rachit

Shkolnikov, Vladimir

Siddiqui, Muhammad

Silva, Diego

Silva, Maria-Raquel G.

Sin, May

Singh, Jerome

Soares, Halley

Soliman, Marco

Soma-Pillay, Priya

Sonderup, Mark

Spiegel, David

Spiegel, Paul

Sra, Manraj

Stavrinaki, Tina

Steel, Amie

Steinmann, Peter

Stites, Shana

Stivas, Dionysios

Suelves, Josep M.

Suleman, Fatima

Sullivan, David

Sun, Qiang

Sunderland, Vivian

Sy, Deborah

## T

Taba, Makomane

Taylor, Melanie

ten Brink, Debra

Terrones-Lozano, Alejandro

Thi Hai Van, Hoang

Thillaigovindan, Parivallal

Thompson, Jacqueline

Thu Trang, Vu Thi

Torgerson, Paul

Tran, Anh

Trehan, Indi

Tsai, Feng-jen

Tucker, Joseph

Turbow, Sara

## V

Valentine, Nicole

van Hoof, Joost

Van Truong, Le

Vázquez Torres, Susana

Velayudhan, Raman

Verma, Ajay

Vicario-Merino, Angel

Viney, Ian

Virgili, Gianni

Vojnov, Lara

## W

Walker, Richard

Wang, Hongting

Watanabe, Chiho

Weerasuriya, Krisantha

Weldon, Isaac

Wibuloutai, Jindawan

Wiesner, Jacqueline

Wild, Hannah

Willis, Matthew

Winter, Sebastian

Witt, Clara

Wong, Brad

Wong, MS

Wong, Vincent

Woodward, Alistair

Wozniak, Gregory

Wozniak, Teresa

Wright, Stephen

Wushouer, Haishaerjiang

## X

Xiang, Xin

Xiao, Bin

Xu, Hongwei

## Y

Yachan, Li

Yates, Tom

Yatsuya, Hiroshi

Yew, Ying Ying

Yisa, Abraham

## Z

Zakeri, Marjan

Zhang, Xiaohui

Zhang, Xu-Sheng

Zheng, Weiwei

Zheng, Yunting

Zhou, Chengchao

Zhuo Lin, Chong

Zicker, Fabio

Zisovska, Elizabeta

